# How Do Low-Income Urban African Americans and Latinos Feel about Telemedicine? A Diffusion of Innovation Analysis

**DOI:** 10.1155/2012/715194

**Published:** 2012-09-10

**Authors:** Sheba George, Alison Hamilton, Richard S. Baker

**Affiliations:** ^1^Center for Biomedical Informatics, Charles R. Drew University of Medicine and Science, 2594 Industry Way, Lynwood, CA 90262, USA; ^2^Department of Psychiatry, UCLA and VA Greater Los Angeles Healthcare System, 11301 Wilshire Boulevard, Los Angeles, CA 90073, USA; ^3^College of Medicine, Charles R. Drew University of Medicine and Science, 1731 E. 120th Street, Los Angeles, CA 90059, USA

## Abstract

*Introduction*. Telemedicine is promoted as a means to increase access to specialty medical care among the urban underserved, yet little is known about its acceptability among these populations. We used components of a diffusion of innovation conceptual framework to analyze preexperience perceptions about telemedicine to assess its appeal among urban underserved African Americans and Latinos. *Methods*. Ten focus groups were conducted with African American (*n* = 43) and Latino participants (*n* = 44) in both English and Spanish and analyzed for key themes. *Results*. Both groups perceived increased and immediate access to multiple medical opinions and reduced wait time as relative advantages of telemedicine. However, African Americans expressed more concerns than Latinos about confidentiality, privacy, and the physical absence of the specialist. This difference may reflect lower levels of trust in new health care innovations among African Americans resulting from a legacy of past abuses in the US medical system as compared to immigrant Latinos who do not have this particular historical backdrop. *Conclusions*. These findings have implications for important issues such as adoption of telemedicine, patient satisfaction, doctor-patient interactions, and the development and tailoring of strategies targeted to each of these populations for the introduction, marketing, and implementation of telemedicine.

## 1. Introduction

Telemedicine involves using computer information and telecommunication technologies to provide health care when the provider and care recipient are in separate geographic locations. It has been promoted as a vehicle to increase access to specialty care among the urban underserved minorities, yet little is known about its acceptability among such populations. The literature on the adoption and diffusion of new technology, such as telemedicine, suggests that stakeholders' perceptions about a new innovation and the extent to which they see it as a “relative advantage” are central to the rate of diffusion and adoption [1]. The objective of this study is to explore perceptions regarding telemedicine among African Americans and Latinos in South Central Los Angeles.

It is well documented that racial/ethnic minorities and socioeconomically disadvantaged individuals face significant barriers to receiving basic health care [2–6]. African Americans and Latinos make up the largest proportion of minority populations who experience the most severe and concentrated types of health disparities. Much of this disparity in health is thought to be due to lack of timely access to appropriate health care [3]. Medically underserved populations experiencing health disparities tend to be concentrated in either inner city or rural areas. These areas are plagued by low physician-to-population ratios, limited specialty care, and health care facilities that suffer from overcrowding, inadequate infrastructure, and inefficient organizational structures [2–5, 7–11]. Given that the Institute of Medicine's report on quality of health care has already identified illiteracy and distrust of technology as potential barriers to the delivery of telemedicine in urban underserved settings, it is important to assess community perceptions of this technology [8]. South Central Los Angeles serves as a prime example of such an inner city setting, making it an excellent location for a case study.

 Telemedicine has been promoted as an innovative approach to bridging the health care delivery gap by increasing access to services for medically underserved communities. The role of telemedicine in facilitating increased access to care has traditionally been framed in terms of its ability to mitigate geographic barriers. Accordingly, remote rural communities have been the primary beneficiaries of telemedicine implementation [12]. However, limited access to appropriate medical care, particularly specialty care, is a major challenge for inner city communities as well. 

Although telemedicine has the potential of redressing the health care delivery problems of the inner city, there is little in the existing literature on telemedicine or health care in general that sheds light on perceptions about telemedicine among the general population and, more specifically, the urban underserved population [9, 10, 13, 14]. It is important to examine the concurrence (or divergence) between the medical aims that drive such solutions and “on-the-ground” perceptions of those receiving care, particularly among inner city African American and Latino populations. In a study of an urban urgent care dermatology clinic, while patients generally reported high levels of satisfaction, 36% of the study sample expressed self-consciousness around the camera and 17% were uncomfortable having facial pictures taken [15]. In terms of outcomes, the Informatics for Diabetes Education and Telemedicine (IDEATel) project [5] found that African American and Hispanic American participants were less adherent to the diabetes self-care intervention than white participants, suggesting the need for culturally tailored interventions [16, 17]. The issue of community acceptance of such new techniques has yet to be resolved. 

Diffusion of innovation (DOI) theory is useful in understanding the importance of assessing perceptions about a new technology such as telemedicine among a population before its introduction in order to promote likelihood of adoption. Though there are several components to DOI theory, here we focus on its applicability to characteristics of the innovation itself, that is, how an innovation spreads from innovators to others within a social system. Rogers' classic DOI model points to five factors that shape the rate of diffusion of new innovations among stakeholders: (a) the perception of relative advantages, (b) the compatibility with past experiences and existing values, (c) the complexity of the innovation, (d) observability of benefits, and (e) trialability of the innovation on a limited basis. For example, according to this framework, if patients perceive the relative advantages of using telemedicine to be greater than existing options with regard to savings in time/money, increases in comfort, social status, and so forth, they will be more likely to adopt telemedicine. The compatibility factor points to the importance of consistency of telemedicine use with “past experiences, existing values and needs of potential adopters” [18]. Whereas the first two factors focus on the stakeholders' needs, experiences, and values, the latter three factors (complexity, observability of benefits, and trialability) focus on aspects of the innovation. It seems important to understand which of these factors maybe most relevant at baseline for specific type of populations within particular geographical contexts vis-à-vis a new innovation in order to best promote the diffusion of the innovation.

 Most of the studies that examine patient perceptions about telemedicine tend to question participants on their past experiences of receiving health care through telemedicine [19, 20]. However, preexperience perceptions are important to the success of telemedicine adoption since they shape a patient's initial decision to (a) sample a telemedicine service and (b) use the service on a continual basis [21]. There is scant research on viewpoints about telemedicine among the target population before the introduction of telemedicine. Some exceptions include studies by Bashshur [22], Brick et al. [23], and Turner et al. [21]. The first two studies found that patients do not perceive telemedicine as preferable to seeing a doctor in person, even though they appreciated the usefulness of telemedicine for emergency situations and minor problems. Turner and colleagues found that the greater the perceived relative advantage and the greater the perceived compatibility of the innovation, the greater the intent to adopt it with varying levels of openness depending on the task situation (e.g., their respondents were more open to telemedicine care in emergency situations than for specialist care). However, none of these studies examines the perceptions of urban inner-city populations regarding telemedicine and the specificities of their care contexts.

Given that there is little research on the perceptions about telemedicine among African American and Latino underserved populations, we examined the pretelemedicine perceptions of these groups and the differences between them. In addition to our focus on these two populations, we were interested in identifying the differences between elders in these groups (over 65) and younger adults (parents of school-aged children) since these are the two groups most likely to utilize and benefit from telemedicine services with a clear source of health care reimbursement. We hypothesized that the elderly would be less amenable to the idea of new technology and parents would be more willing to consider trying the technology to meet the needs of their children.

## 2. Methods

### 2.1. Setting: South Central Los Angeles

The research was conducted in South Central Los Angeles, which is home to more than 1.4 million individuals, most of whom are racial/ethnic minorities (62.7% Hispanic, 33.4% African American). South Central is the most socioeconomically disadvantaged community in Los Angeles, with 28% of the population living below the federal poverty level [24]. The population faces several barriers to receiving timely care: in 2005, 40.2% reported that they could not afford to see a physician when needed and 27.5% of adults reported transportation problems as a barrier that kept them from obtaining needed medical care [25].

### 2.2. Procedures

Focus group methodology was utilized to explore the range of individual opinions within relatively homogeneous groups (described below), using a standardized set of questions [26–29]. The research team consisted of the authors and two research associates who assisted with the recruitment and moderation of the focus groups.

Community-based recruiting efforts were used to develop a sample population for the focus groups. Flyers about the focus groups were posted in community centers and public housing complexes. Interested individuals called the number on the flyer. When 8–10 individuals from the priority populations (African American and Latino parents of school-aged children and seniors) responded to these efforts, focus groups were assembled (see Table 1 for group composition). The focus groups took place in community and senior centers. Informed consent was obtained from all participants. All participants completed a background questionnaire and were paid $20 at the end of the focus group. 

After introductions, participants were asked for their definitions of the word “telemedicine.” After a short discussion, a brief video presentation—a dramatization of a patient, receiving care for ear pain at a telemedicine clinic—was shown to focus group participants. Groups that were Spanish speaking (5 of the 10 groups) were shown a Spanish version of the video. In the video, the patient's ear pain is assessed by a physician's assistant (PA) who contacts an ear, nose and throat (ENT) specialist using videoconferencing. This ENT specialist is depicted as being distant from the clinic; he examines the patient using an otoscope with a camera at the end, which transmits images of the patient's ear to the specialist. All parties (patient, specialist, PA) are able to see each other through videoconferencing technology. 

 The video was followed by a focus group discussion about participants' reactions to and perceptions about receiving medical care through telemedicine. The moderator used a semistructured interview script that covered reactions to the video, perceived advantages and disadvantages of telemedicine, diagnoses/health conditions for which telemedicine would be appropriate, and general experiences in receiving health care services (Table 2). 

### 2.3. Data Analysis

All interviews were audio- and videotaped and transcribed, and all Spanish-language transcripts were translated into English by a professional transcription and translation agency. Atlas, it was used for data analysis. The transcripts were analyzed using the constant comparative method of data analysis [30]. Transcripts were initially deductively coded by the second author with questions from the interview script guiding the predominant themes. These themes were summarized and discussed by the research team, and then the data underwent another level of more inductive coding to explicate the range of issues that were raised in response to each question and to compare across categorical groupings (parents versus elders, African American participants versus Latino participants). Through an iterative process of immersion in the data and refining the categories, key themes and DOI theoretical insights were identified and interpreted collaboratively by the authors.

## 3. Results

Participants emphasized two DOI factors: relative advantages and compatibility with experiences and existing values. There were some differences between African Americans and Latinos in how they viewed these factors. While the two groups had similar perceptions of the relative advantages of telemedicine, they had differing perceptions of the compatibility of telemedicine with their experiences and existing values, resulting in different types of concerns (see Table 3). Participants were understandably less prone to raise innovation-focused factors that are important to rate of adoption (complexity, observability of benefits, and trialability) because they were not familiar with telemedicine. We did not identify consistent discernible differences by age.

### 3.1. Relative Advantages

For both African Americans and Latinos, there were several relative advantages to telemedicine as compared to their usual modes of health care. The main advantages noted in all of the focus groups were: (1) reduced waiting time, (2) immediate feedback as to diagnosis and course of action, (3) increased access to specialists, and (4) increased access to multiple medical opinions. It is important to note that these perceived advantages are not necessarily correct perceptions of how telemedicine operates, but they do illustrate the values that participants associated with this type of system. 

 With regard to speed and accuracy of diagnosis, one Latino participant said that telemedicine would be a “novelty” because “it can give you the diagnosis right away cause they' re consulting the specialist so you can get your diagnosis instantly. I think that's good.” Another Latino participant in another group said: “Science is more advanced and you will be able to see everything through the Internet … It will be like having the doctor in front of you but you won't have to go to his office. The laboratory won't take a lot of time and you will really know what you have.” 

 Telemedicine's potential convenience in terms of these issues and in terms of logistics (such as location) was perceived to be very appealing. The African American participants felt that telemedicine would be particularly beneficial for children and the elderly. For example, one participant said, “I can see it going places. I can see where people will like it. Young people will love it. Their families, I can see my children, you know, loving it for their children, you know, in many cases. First, because they do not have enough time to do whatever, you know, because they are so busy all the time. So that helps to get an immediate feedback and to give a diagnosis and a solution to a problem.”

 While the same four major advantages were discussed in all of the groups, Latino participants also noted several additional advantages and seemed, overall, more positive and enthusiastic about the prospect of telemedicine. They felt that telemedicine could potentially cut down on misdiagnoses, particularly because the computer gives “exact data.” This idea of the precision of computers was raised in three of the Latino focus groups. One group felt that telemedicine might result in more choice over which doctor is assessing you and might provide better doctors. One group pointed out that telemedicine would result in more jobs for nurses.

 The location and convenience of the clinics were also discussed more extensively in the Latino focus groups, because they felt that the clinics would be easier with children and with transportation. For example, “I would love something like this to open as soon as possible, because we need it. We need it for all of our children, because sometimes we take them all in when one has an appointment. You save time seeing the specialist that one of your children needs, or if another specialist is needed, you don't waste any time, you save time and see the doctor you want to see and it would be great if Medi-care would pay for these services.” 

#### 3.1.1. Concerns about Compatibility with Past Experiences and Existing Values

African Americans and Latinos had very different perceptions about the compatibility of this innovation with their experiences and existing values. Participants' main concerns about telemedicine were confidentiality/privacy (considering the use of the Internet for the transmission of personal information) and the process of diagnosis (considering the use of scopes rather than actual clinical observation, i.e., physician's physical presence). Overall, African Americans were more concerned about these issues, and were especially concerned about the physical absence of the physician and the perceived inability to monitor the (distant) specialist's qualifications and level of attention. Latino participants were substantially less concerned about these issues and in some cases felt very differently about them. They did, however, express concerns about whether telemedicine would be made accessible to uninsured and undocumented individuals.

#### 3.1.2. Technology Issues: Confidentiality and Privacy

For both African American and Latino participants, the technology critical to telemedicine posed some problems. On a technical level, some participants in both sets of groups discussed the possibility that the computer could go down or the system could fail. More important than this concern, however, was that personal information could be obtained by individuals other than those involved in the telemedicine encounter. For example, there was discussion among African American participants that one's identity could be stolen and that one's pictures would be “floating around.” The Internet was perceived as “insecure” and “for anybody.” One African American participant noted, “Internet is the Internet. So that means your name is out there and whatever your problem is, it's on the Internet. And you know, records are supposed to be a personal thing between you and your doctor, but if it's going to be on the Internet, then it's for anybody.” In one group, participants discussed how they did not even like the idea of Internet banking due to the possibility of a breach in privacy. In another group, a participant imagined that children would be able to see medical images on the computer: “The kids, you know, they go to the library and they say, “mamma, guess what I see?” Now if I'm sick and there's something wrong with me, I don't want the Internet to know, too. No, no, then the whole world will see.” 

In contrast to the African American participants, Latino participants seemed more confident that privacy would be protected, and they were not as concerned that privacy could not be guaranteed. One participant said that the “standard ordinary person” would not even be interested in “anything scientific, and less still related to health.” These participants, for the most part, expressed that maintenance of confidentiality was the physician's responsibility, and that the physician would not risk his license with a questionable system: “I don't think [the doctor] would risk his degree to give out the files of all the patients that are in the computer because he would be responsible.” In another group, participants discussed asking for confidentiality, and they felt that by asking, confidentiality would be assured: “If you tell the person who's going to carry out the treatment that you want confidentiality in your case, I don't think there would be any problem. But you must ask for it. It won't come on its own.” 

For some Latino participants, the technology assured more privacy. One Latino participant stated, “I feel there's more privacy. I really like the idea because the computer gives you exact data. It makes me feel better, you know? “Cause the fact that you're being looked at through the computer, it removes the self-consciousness, shame, or whatever of talking openly to a doctor. Like this, without being face to face, I can say whatever I wanted.” There was some concern about identity theft, but, overall, Latino participants felt confident that transmitted information would remain confidential. As noted above, even for those who were not convinced of the confidentiality, there was typically little concern. One Latino participant said, “It doesn't matter to me that people should see me because the whole world has to know what science is doing.”

#### 3.1.3. Diagnosis and the Physical Absence of the Specialist

One of the main topics addressed in the focus groups was the physical absence of the specialist, which is one of the main distinguishing features of telemedicine. Discussions on this topic revealed the complexity of the doctor-patient interaction, illustrated by the multiple layers of meaning that participants attach to such interactions. Because of the richness of this set of findings, our results regarding the importance of physical presence and touch in telemedicine for these populations will be elaborated upon in a separated publication and here we will provide a summary of the findings. In general, the participants associated the physical presence or absence of the specialist to their (1) satisfaction with a medical encounter, (2) level of assurance that appropriate information was being conveyed, and (3) ability to accurately gauge the reactions of the specialist and monitor the latter's activities. 

 Several of the African American participants' concerns about not being physically with the specialist seemed related to sensory experiences of vision and touch, that is, being unable to “see” the specialist in person and/or not having the specialist touch the patient. For example, the physical absence of the physician was related to concerns about being able to assess if “the truth” was being told to the patient. For others, it was about being able to monitor the activities of the specialist (e.g., “How do I know that the doctor ain't on the other side and he's getting high?”). The importance of the physical presence of the specialist, particularly sight and touch, was often related to the specialist's capacity to make accurate diagnoses. 

The Latino participants seemed less concerned about the physical absence of the physician in the telemedicine clinic. Having the doctor physically present did not equate with better care for these participants, as expressed in such statements as, “It makes no difference having the doctor in front of you.” Participants expressed that even when the doctor is present, they tend to “only ask questions,” whereas it is the nurse who provides care. The doctor “bases his opinions on what the nurse tells him,” so diagnosis could take place just as well from a distance. 

Interestingly, some Latino participants expressed a preference for telemedicine because of the physical absence of the physician. The reasons for this preference seemed to be centered on embarrassment about gender, age, and class differences between the provider and patient. As one participant explained, she preferred gynaecological exams by telemedicine because it would help her avoid in-person interactions with “young, attractive” (male) gynaecologists.

#### 3.1.4. Qualifications and Qualities of the Physicians

As noted above, some African American participants were concerned that the telemedicine physician would not be giving the patient his/her undivided attention. This relates to an issue that came up in several of the focus groups, which is how do you trust in the physician who is not in the room with you? How do you know he is qualified and certified? 

One African American participant wondered how experienced the telemedicine physicians would be: “How many years of experience have they had? You know, some of them might not even have but six months, some might not even have a year. So you have to take all that into consideration because I myself don't want anything that hasn't been in medicine over a year to be looking at me … I still prefer an experienced doctor, whether he's on telemedicine or I see him in person.” There was suspicion that the physician might not be who s/he claims to be, as expressed by an African American participant in the following question: “What is the reassurance that we have that this so-called specialist that's on the screen really is what he's supposed to be?” 

 Latino participants had more discussions about how they know the qualifications of any physicians, telemedicine or not. Most often, knowledge of a physician's quality and qualifications came from the success of the treatment, the physician's interpersonal qualities, or other people's recommendations. For example, “I have been seeing my current doctor for more than seven years, and he gives you the medicine and so you don't have to come back. And that's how we would know if they are good doctors or not.” Another participant responded, “If I go to my doctor, I'm not 100% sure if he is a doctor or not. In terms of whether or not a doctor is good, well, you try him and see. I like the way I was treated.” 

 In two Latino focus groups, participants agreed that one knows of a doctor's quality because “the medicines he gives you do you good.” The participants said that the telemedicine personnel would be responsible for assuring the quality of the physicians: “We are trusting in you like we trust in the clinics we go to. We trust that the doctor we are going to see is really a certified doctor who has gone to school and who knows medicine. I think you must take that risk, for it's the responsibility of those who are in charge of the clinic.” Latino participants also discussed seeking information on their own as to the qualifications of physicians, for example, by looking on the Internet: “All you do is go to a website and all you have to do is fill in the doctor's name and the clinics you've been to. There are many doctors that have done bad things and they are in jail, and their names are not on the list and that's another way to find out if a doctor is good or not.” In general, while both African American and Latino participants shared concerns about the qualifications of the telemedicine physician providing care, the latter tended to think that the risks were not necessarily greater for telemedicine-based physicians as compared to physicians seen in person, and they expressed more trust that the quality of the physicians would have to be acceptable.

## 4. Discussion

Telemedicine has been promoted as an innovative approach to bridging the health care delivery gap particularly for underserved communities. While inner-city minority communities could potentially benefit from this innovation, there is little in the existing literature that speaks to the acceptability of such a solution among minority populations. To the best of our knowledge, this is the first study that explicitly examines perceptions about telemedicine among urban underserved minority populations, although some studies on telemedicine have included minority cultural groups [31] and studies of minority perceptions of health care in general have been done [32].

 Both African American and Latino focus group participants emphasized two DOI factors that shape the rate of diffusion of an innovation: relative advantage and compatibility with past experience. Participants were less likely to discuss complexity, observability of benefits, and trialability of telemedicine, likely because these factors focus on features of the innovation, with which the study participants were not very familiar. We contend that they were more likely to talk about telemedicine's relative advantages and compatibility since these factors were salient to their current concerns about their health care needs, lived experiences, and existing values, and they could be discussed despite their lack of first-hand experience with telemedicine. 

 The advantages of any health care innovation are usually assessed by potential users relative to their current experiences of receiving care. This was true regarding telemedicine for the focus group participants. Given their underserved inner-city location, the study participants overwhelmingly identified timely access to care as one of the greatest relative advantages of telemedicine. Telemedicine appears to provide some relatively efficient solutions to issues such as the challenge of transportation to get to specialist care, lack of timely access to specialists, the lack of timely diagnoses and feedback, and the lack of multiple opinions in a specialist-scarce zone. 

 However, the two groups had different concerns about health care received through telemedicine, reflecting differences in the compatibility of their lived experiences and values with the perceived nature of telemedicine-based care. For African Americans, their experiences as a community with a history of slavery and continuing racism in many aspects of their lives, particularly with health care, may affect their views on new and innovative medical care [33, 34]. The legacy of past abuses such as medical experimentation on slaves and the Tuskegee syphilis experiment and other types of continuing racism in health care contribute to lower levels of trust and a higher level of suspicion [34–37]. 

A related issue that has been studied in more detail is the attitude of minorities toward enrollment in medical research, where similar findings have been reported about African American attitudes towards research [38–46]. Among African Americans, mistrust is frequently associated with the perception that research will benefit whites or the research institution and not people of color. Furthermore, mistrust of the health care system was a primary barrier that prevented African Americans from participating in medical research [38]. 

 For the African American participants in this study, the emphatic need to “see” and “touch” the physician seemed related to similar issues of trust. The physical absence of the physician, the instability of technology, and the inability to monitor the specialist's qualifications were all highlighted as concerns with telemedicine for the African American participants. All these concerns reflect a sense of vulnerability when placing trust in a medical system that historically has been unreliable and not trustworthy. African American participants expressed a need to be vigilant and monitor physicians to make sure that they would get quality care, particularly when telemedicine appeared to present greater opportunities for care to be compromised. This concern about quality of care is consistent with literature that indicates African Americans' less than satisfying interactions with physicians [47, 48]. 

With regard to technology, there were many levels of concern. First, there was concern about whether the scopes used in telemedicine would perform adequately to allow physicians to make accurate diagnoses. Second, there was some concern about the computer system failing. However, the bulk of the apprehension among African Americans regarding technology was about the insecurity of transmitting personal data and images over the Internet when using telemedicine. A third issue of trust reiterated by African American participants was that of being able to trust the qualifications and qualities of the physician who is not in the room. There was concern about the level of experience of the physician, suggesting that these participants were concerned that telemedicine might be a way to unload inexperienced or second-rate doctors on them. 

 In contrast, the Latino participants had distinctly different responses to telemedicine, which may be explained partly by their different vantage points and lived experiences. Latinos, across age groups, appeared to have a significantly more trusting attitude towards the health care system in general and telemedicine in particular. This difference was reflected in their very different attitudes towards the telemedicine-related issues identified as problematic by African Americans, namely, the physician's virtual presence, the usage of technology, and the qualifications and qualities of physicians. The Latino participants' relative lack of concern about the physical absence of the physician points to the possibility that physical exams and the touch of the physician in time-pressured primary care visits are becoming less frequent [49] and consequently telemedicine is not that different from their expected standards of care. 

Latino participants tended to equate the use of technology with access to scientific advances and expressed faith in the appropriate authorities to maintain confidentiality. Technology was seen by many Latinos as assuring greater accuracy (more exact data). Such optimism and openness towards technological innovations among Latinos was markedly different from the attitude found among African American participants. Despite the fact that both groups may experience what is commonly called the “digital divide,” they had noticeably different opinions about technology in general.

 Latino participants also differed from African Americans in that they trusted the administrators of both telemedicine and non-telemedicine clinics to be responsible for hiring qualified doctors. Finally, the knowledge of the quality and qualifications of the physicians was determined by the success of the treatment, whether telemedicine or nontelemedicine based.

 The qualitative racial/ethnic differences in attitudes about telemedicine-based health care among Latinos and African Americans point to differences in their lived experiences and values. The point of reference for many African Americans is the history of racism and medical experimentation and abuse they have experienced collectively in the United States. In contrast, immigrant Latinos encounter the US medical system without this particular historical backdrop and their point of reference maybe less than optimal health care in their home countries, along with a generally positive perception of the American health care and medical education systems. For many of the immigrant Latinos, access to American health care and especially telemedicine-based care that is perceived as scientifically and technologically cutting edge also seems to be seen as a positive improvement. Thus, in terms of the DOI framework, there appears to be good compatibility between the needs, lived experiences, and values of Latinos with the structure and delivery of telemedicine-based care.

### 4.1. Implications for Telemedicine

Our findings of differences in attitudes toward telemedicine suggest that it will be necessary to tailor approaches to the introduction, marketing, and implementation of telemedicine among these different populations. It is critical to gather this information before the extensive introduction of telemedicine clinics in inner-city communities for at least three reasons. 

 First, this information can be important for determining the best manner in which to introduce and market telemedicine among these two groups. Based on the findings from this study, it is important to identify the gaps in knowledge or the misinformation that can lead to distrust of new technology or the overestimation about the benefits of new technology and false expectations. The information gathered from this study can be used to help lower the barriers to acceptance of telemedicine by developing educational materials that address misinformation and gaps in knowledge. Marketing information could be tailored to address the specific concerns voiced by the two racial/ethnic groups, such as clearly informing African Americans about the medical qualifications of the specialists and the security procedures for maintaining confidentiality and level of diagnostic accuracy using telemedicine equipment. 

 Second, this information can be important in selecting the optimal ways in which to implement new telemedicine clinics. For example, for African Americans, having an initial in-person meeting with a physician may be important in helping establish trust and better preparing the patient for future virtual appointments. For real-time telemedicine consultations, cameras could be set up to make the specialist's activities especially transparent to the patients. Physicians' assistants or the nurses in the clinics and the specialists involved in telemedicine could be better informed about the concerns of each of these groups so that they can address these concerns (such as reassurances about confidentiality), even if the patients do not voice them.

 Third, this data can also serve as a baseline point of comparison for studies that will examine changes in patient perceptions over time. As telemedicine becomes implemented in urban settings and becomes more familiar to African American and Latino populations, it will be important to have an understanding of their baseline pre-experience perceptions regarding telemedicine to gauge the changes in attitudes towards telemedicine as it spreads into different communities. 

### 4.2. Limitations

There are several important limitations to our data and study findings. First, we have a relatively small convenience sample and our participants are not statistically representative of the wider population in inner-city settings. However, as is common to qualitative methods, they represent information-rich cases, homogenously stratified across race and age, to allow in-depth understanding of the perceptions about telemedicine among these groups. Another limitation is that for the majority of our participants, the only information about telemedicine came from the video they saw at the beginning of the focus group. While telemedicine was portrayed in a typical setting with a typical health problem, our participants' understanding and consequent reactions to telemedicine were clearly influenced by what we were able to show them in a short video. For example, we represented telemedicine primarily as a diagnostic interaction with a specialist and did not address its other potential uses, such as in the long-term management of chronic diseases. Our finding of no age group differences may be a reflection of the limitations of our study design. We may have needed a more sensitive interview protocol that would have more finely delineated the nuances of age differences in our sample. 

## 5. Conclusion

Using the DOI framework regarding features of an innovation, this study contributes to an underresearched area by exploring the pre-experience perceptions of telemedicine among urban, underserved African Americans and Latinos. Despite reservations, many participants indicated that they would take advantage of telemedicine clinics. 

Through this study, we were able to identify components of the DOI framework that spoke to the experiences of the two minority groups—particularly with regards to compatibility with past experiences and existing values. It will be important to develop larger studies in different geographical regions with different populations to further understand the importance of these factors for the introduction/marketing, implementation, and eventual adoption of telemedicine among diverse populations.

## Figures and Tables

**Table 1:** Focus group composition.

	African Americans *N* = 43		Latinos *N* = 44	
	Groups	*N*	Groups	*N*
Seniors, *N* = 37 (average age = 67; range 61–83 years)	1	9	6	10
	2	8	7	10

Parents, *N* = 50 (average age = 34; range 21–55 years)	3	7	8	8
	4	9	9	9
	5	10	10	7

**Table 2:** Focus group script—interview themes and examples of questions*.

Broad themes	Example questions
A telemedicine clinic in your community	(i) How do you feel about it?
	(ii) How did you form this impression?
	(iii) From what particular experiences?

Perceived advantages and disadvantages of telemedicine	(i) What are specific benefits?
	(ii) What are potential challenges?
	(iii) Would telemedicine address any specific gaps/issues you have with your present form of health care?

Ideal recipients of telemedicine care	(i) Would you use telemedicine yourself?
	(ii) Would you recommend it to a friend?
	(iii) Would it be particularly suitable for older people/young children?

Conditions and context of use	(i) For what types of health conditions would you be most comfortable using telemedicine?
	(ii) How often and under what conditions (e.g., weekends only) would you want to use such a clinic?

^∗^These are examples of only some of the initiating questions used. Other more probing questions were asked of participants depending on what their responses were in order to gain more in-depth information.

**Table 3:** Advantages and concerns about telemedicine for African American and Latino participants.

	Advantages	Concerns
African Americans	(1) Reduced waiting time (2) Immediate feedback (3) Increased access to specialists (4) Increased access to multiple medical opinions (5) Convenience for children and the elderly	(1) The physical absence of the physician specialist (2) Ability to monitor the specialist's qualifications (3) Privacy/confidentiality issues related to the presence of personal information on the Internet (4) Adequacy of telemedicine scopes to make accurate diagnoses

Latinos	(1) Reduced waiting time (2) Immediate feedback (3) Increased access to specialists (4) Increased access to multiple medical opinions (5) Convenience for children and the elderly (6) Greater accuracy of diagnoses due to precision of computers (7) Avoiding poverty-related embarrassment and in-person physician interaction	(1) Privacy/confidentiality issues related to the presence of personal information on the internet, to a lesser extent (2) Adequacy of telemedicine scopes to make accurate diagnoses, to a lesser extent (3) Concerns about whether telemedicine would be available to uninsured/undocumented
